# A heterodox defense of the actualist higher-order thought theory

**DOI:** 10.1007/s11098-021-01726-w

**Published:** 2021-09-09

**Authors:** Andrea Marchesi

**Affiliations:** Department of Philosophy (KGW), University of Salzburg, Franziskanergasse 1, 5020 Salzburg, Austria

**Keywords:** Consciousness, Higher-order thoughts, Circularity, Evidence, Introspection, Intimacy, David Rosenthal

## Abstract

I defend the actualist higher-order thought theory against four objections. The first objection contends that the theory is circular. The second one contends that the theory is unable to account for the alleged epistemic position we are in with respect to our own conscious mental states. The third one contends that the theory is unable to account for the evidence we have for the proposition that all conscious mental states are represented. The fourth one contends that the theory does not accommodate the intimacy we have with our own conscious mental states. To some extent, my defense will be heterodox, in the sense that I will show that some objections are satisfactorily answerable even if we concede to the objectors a point that higher-order theorists do not seem to be willing to concede, that is, that the theory is the result of conceptual analysis.

## Introduction

Higher-order theories of consciousness have an almost paradoxical status: they are inspired by a principle that sounds offhand plausible, but they have also been bombarded with a volley of objections.^1^ The relevant principle is the so-called *transitivity principle* (see Rosenthal, 2000): it says that conscious mental states are states we are conscious of. Starting from this “insight,” as it is often referred to, higher-order theorists have developed different and competing accounts of what it is for a mental state to be a conscious state. Given the high level of refinement that these accounts have reached, and given the high number of attacks that have been made on them, it is useful to follow Carruthers (2016) in distinguishing between *generic* and *local* objections: the first ones are those leveled at higher-order theories *as such*; the second ones are those leveled at *this or that* variant of higher-order theories.

In this paper, I will defend a particular variant of the higher-order theory against a limited number of objections. The higher-order theory I will defend is the *actualist higher-order thought theory*, in its original formulation. Roughly, such theory has it that our mental states are conscious just in case we have an assertoric thought about them. The most prominent advocate of this theory is David Rosenthal. The local objections I will try to disarm are four. I will refer to them as the *circularity objection*, the *first epistemic objection*, the *second epistemic objection*, and the *intimacy objection* respectively. The circularity objection, raised by Rowlands (2001), contends that the theory is circular at its core. The first epistemic objection, raised by Goldman (2002), contends that the theory is unable to accommodate the alleged evidence we automatically have when our states are conscious. The second epistemic objection, raised by Kriegel (2009a, b), contends that the theory cannot account for the evidence we have for a certain (universal) proposition about consciousness. The intimacy objection, raised by several scholars, contends that the theory does not accommodate the intimacy we have with our own conscious mental states.^2^

All the foregoing objections have been already explicitly addressed: the first two objections have been dealt with by Carruthers (2016), the second epistemic objection has been appraised by Levine (2010) and van Gulick (2012), and the intimacy objection has been recently scrutinized by Rosenthal (2018) himself. Nevertheless, I believe that there is still room for an assessment. Concerning the objections raised by Goldman and Rowlands, I will argue that they are satisfactorily answerable even if we concede to the objectors a point that Carruthers and other higher-order theorists do not seem willing to concede, that is, that theory is the result of *conceptual* analysis. In this sense, my defense will be heterodox. Akin considerations apply to my defense against the intimacy objection: I will argue that Rosenthal’s reply is deficient and put forward a refined one. Concerning the second epistemic objection, the existing replies are prior to Kriegel’s (2012) prompt counter-response, which has not yet been assessed and is worth considering.

Before beginning a remark is in order. Recently, friends of the actualist HOT theory have proposed to construe it with a novel form, especially because they have taken the possibility of false higher-order thoughts to cause trouble. The novel formulation is commonly labeled as the “non-relational” formulation of the actualist HOT theory (see notably Berger, 2014; Brown, 2015; Gottlieb forthcoming) and is contrasted with the original formulation (see Carruthers, 2016), which is labeled—not uncontroversially—as “relational.”^3^ Roughly, on the novel formulation, the theory turns out to be a theory of *creature* consciousness,^4^ whereas on the original formulation the theory is a theory of *state* consciousness (I will clarify these notions along the way). As I said, I will defend the theory in its original formulation. I believe that such a defense is worth making for two reasons. The first one, that I cannot develop here for reasons of space, is that the conclusion that the possibility of false higher-order thoughts causes trouble for the original formulation of the theory can be disputed.^5^ The second one is that the original formulation is the formulation that the creator of the theory—David Rosenthal—still holds. With his more recent words:

Being aware of a state is necessary for that state to be conscious, but it is not sufficient. Still, we can begin to close in on a sufficient condition by determining how one must be aware of a state for that state to be conscious. I’ve argued elsewhere […] that the required type of awareness consists in one’s having a thought that one is in that state. (Rosenthal forthcoming)

The architecture of the paper is as follows. Section 2 presents the basics of the actualist HOT theory and briefly discusses its nature. Section 3 is devoted to the circularity objection. Sections 4 and 5 are devoted to the two epistemic objections. Section 6 is devoted to the intimacy objection. The final section sums up the results.

## The actualist higher-order thought theory

We use the predicate “conscious” to denote different properties. Accordingly, we have to make some preliminary distinctions. First, we may ascribe consciousness both to creatures (individuals) and mental states: *creature* consciousness is the property of a creature’s being conscious; *state* consciousness is the property of a state’s being conscious. Consider the following two sentences:

1. Mary is conscious.
2. Mary’s thought about her son is conscious.

While (1) expresses creature consciousness (it means that Mary is awake), (2) expresses state consciousness (it means that Mary is aware that she is thinking about her son).

Second, creature consciousness admits two variants (see Rosenthal, 1986): *intransitive* consciousness is the property of being conscious; *transitive* consciousness is the property of being conscious of something. Consider the following three sentences:

1. Mary is conscious.
2. Mary’s thought about her son is conscious.
3. Mary is conscious of her own thought about her son.

While (1) and (2) express intransitive consciousness, (3) expresses transitive consciousness. Note that while we may ascribe intransitive consciousness both to creatures and mental states, we may not ascribe transitive consciousness to mental states. Consider:

(4) Mary’s thought is conscious of her son.

Sentences like (4) are ungrammatical (or at least nonsensical; see Kriegel, 2009b, p. 27).

How do higher-order theories of consciousness account for state consciousness?^6^ First, higher-order theories explain state consciousness in terms of *transitive* consciousness. These theories are centered on the so-called transitivity principle, which can be put as follows: if S’s M is conscious, then S is conscious of M*—*where “S” stands for “subject” (or creature) and “M” stands for “mental state” (here and henceforth). Accordingly, our conscious mental states are those states we are conscious of, whereas our unconscious mental states are those states we are not conscious of.

Second, higher-order theories explain transitive consciousness in terms of *representation*. They say that if *x* is conscious of *y*, then *x* represents *y*. Representations may be of different sorts. Higher-order theorists disagree on what sort of representation is the higher-order one: according to the higher-order *perception* theory, the representation which makes a state conscious is perception-like (see, e.g., Lycan, 1996), whereas according to the higher-order *thought* theory, the representation which makes a state conscious is a thought (see, e.g., Rosenthal, 1997).

Finally, the higher-order thought (HOT) theory branches out into two variants. The *actualist* HOT theory says that S’s M is conscious if and only if S *has* a thought to the effect that S is in M. The *dispositionalist* HOT theory says that S’s M is conscious if and only if S *is disposed to have* a thought to the effect that S is in M. Call the foregoing formulation of the actualist HOT theory the *core claim* of the actualist HOT theorist.^7^

The actualist HOT theorist also commits herself to claims that do not follow from the core claim. Indeed, she not only say that (a) what makes a mental state M conscious is that M is the object of *a mental state* (to be precise, a thought) but also what follows: (b) the higher-order state can be (and normally is) *unconscious* and (c) the higher-order state neither *is identical* nor *belongs* to the lower-order state. All these claims have been seen as breeding infelicitous consequences for the actualist HOT theory: (a) is regarded by Rowlands (2001) as what renders the actualist HOT theory circular. (b) is taken by Goldman (2002) and Kriegel (2009a, b) as what makes the relevant theory unsatisfactory. Goldman argues that (b) makes the theory unable to account for the epistemic position we are in with respect to our own conscious mental states; Kriegel argues that (b) makes the relevant theory unable to account for the evidence we have for the proposition that all conscious mental states are represented. As for (c), it is considered by many scholars the reason why the theory does not accommodate the intimacy we have with our own conscious mental states.

To deal with these critiques, as well as with the replies that have been given to them, one has to consider the following question: what kind of theory is the actualist HOT theory? According to Byrne (1997, p. 103), any higher-order theory is meant to provide a *reductive* analysis of state consciousness, that is: any higher-order theory aims to provide an account that gives necessary and sufficient conditions for a mental state to be conscious that do not presuppose the notion of state consciousness.^8^ Rowlands (2001, p. 291) and Goldman (2002, p. 117) agree, but ask a question: is the actualist HOT theory the result of *conceptual* analysis, or is it something else? Both suggest that there are strong reasons to presume that the actualist HOT theory is, at least partly, a result of conceptual analysis. Rowlands (2001, p. 295) draws our attention to the fact that in setting out the theory Rosenthal explicitly tries to safeguard it against triviality (or better, circularity), whereas Goldman (2002, p. 134 n2) draws our attention to the way the theory is formulated, which according to him makes it sound close to a definition.

What about Rosenthal’s stance? He explicitly denies that his theory expresses a conceptual truth. He describes the actualist HOT theory not as a definition, but rather as a *hypothesis*. In his words:

Since the theory relies for support on its explanatory advantages, it doesn’t appeal to, nor is it intended to reflect, any conceptual or metaphysically necessary truths. The claim that conscious mental states are states we’re conscious of ourselves as being in by having HOTs [higher-order thoughts] about them is a theoretical claim justified by its explanatory power, not a metaphysical truth or a result of conceptual analysis. (Rosenthal, 2005, p. 9)

Yet, I think that the Rowlands-Goldman reading has to be taken seriously. First, because it does not make sense to try to safeguard a theory against circularity—as Rosenthal in fact does—if that theory is not even partly conceptual. Second, because it is not entirely clear what a “theoretical claim justified by its explanatory power” would be. At any rate, I will limit myself to arguing along the following lines: *even if* the actualist HOT theory were the result of conceptual analysis, it would still be immune to the objections raised by Rowlands and Goldman.

## The circularity objection

We know that higher-order theorists try to explain state consciousness in terms of transitive consciousness. As they use consciousness to explain consciousness, one could object that such theories are circular.^9^ However, the higher-order theorist can draft the following rejoinder: the consciousness that figures in the *explanans* is *not* the consciousness that figures in the *explanandum*. He can argue for this difference by pointing out, for instance, that while intransitive consciousness applies both to creatures and mental states, transitive consciousness applies only to creatures. Still, one could reply that circularity is just around the corner. Consider again the core claim of the actualist HOT theory: S’s M is conscious if and only if S has a thought to the effect that S is in M. As it can be seen, the actualist HOT theory explains state consciousness by invoking *mental states*. More precisely: it explains state consciousness in terms of a relation that a mental state bears to another one.

Rosenthal (1997, p. 735) is perfectly aware of the danger. In this regard, he says that if all states were conscious, then the actualist HOT theory would be circular. Is he right about that? It is not obvious: if one takes both “all states are conscious” and “a conscious mental state is a state that is the object of a mental state” to be *conceptual* truths (by modeling the first one on “all bachelor are unmarried”), then one may conclude that the actualist HOT theory is circular, for on this view the concept of mental state entails the concept of state consciousness. This would be expressed by “if *x* is mental, then *x* is conscious” or by “mental states are essentially conscious.” By contrast, if one does not take “all states are conscious” to be a conceptual truth, then there is still room for avoiding circularity. Indeed, one could hold “all states are conscious” to be a result of observation (by modeling it on “all ravens are black”), and if the claim that all states are conscious is made on such a basis, then one will face a problem of *infinite regress* (of mental states), not a problem of circularity.

Either way, Rosenthal is not in trouble, for he holds that *not* all states are conscious. As Carruthers (2016) aptly remarks, this is one of the main motivations of the higher-order theory. However, one could point out that the actualist HOT theorist needs something more to avoid circularity. Indeed, she must deny that both of the following theses express a conceptual truth:

(i) All *states* are conscious.
(ii) All *thoughts* are conscious.

For if one takes both “all thoughts are conscious” and “a conscious mental state is a state that is the object of a thought” to be conceptual truths, then one still faces circularity, since on this view the concept of thought entails the concept of state consciousness (this would be expressed by “if *x* is a thought, then *x* is conscious” or by “thoughts are essentially conscious”).^10^ Yet, one can define thought without referring to state consciousness.^11^ As Rosenthal denies that (i) and (ii) are conceptually true, one could say that the actualist HOT theory is immune to the circularity objection.

Rowlands, though, contends that in order to avoid circularity we need even more. In his words, one needs to establish that “there are no logical connections at all between mental states and consciousness.” What does he mean by “logical connection”? His answer is as follows: if X is logically connected to Y, then the concept of X “entails the concept” (Rowlands, 2001, p. 296) of Y. Alternatively: if X is logically connected to Y, then the understanding of the concept of X “requires the understanding of the concept” (Rowlands, 2001, p. 297) of Y. According to Rowlands, not only are there “logical connections” between *higher-order thoughts* and state consciousness but there are also logical connections between *mental states* and state consciousness. This, coupled with the highly plausible thesis that logical connections are transitive, would make the actualist HOT theory a circular theory. But what would these “logical connections” be? Rowlands writes:

It might be claimed that while no mental states are essentially conscious, nevertheless all mental states are essentially such that they *could* become conscious. Or, we might weaken this still further and claim that while no mental states are essentially conscious, and while at least some mental states are such that they could never become conscious, nevertheless, for any token mental state M, M is a token of the same type as other tokens which are or could become conscious. […] Admittedly, this connection is not as straightforward as the implausible ‘all mental states must be conscious’ variety. But it is a logical connection nonetheless, and thus threatens the HOT model with circularity no less than the more straightforward version. (Rowlands, 2001, pp. 297–298)

In this context, we may replace “could become” with “can be.” Thus, according to Rowlands the “logical connection” between mental states and state consciousness is not only expressed by the thesis that all states are conscious, but also by the following further thesis:

(iii) All states *can be* conscious.^12^

And this is a thesis that Rosenthal does *not* deny.

Carruthers (2016) tries to disarm the relevant objection right away. For him, the objection fails simply because the actualist HOT theory is *not* the result of conceptual analysis. Is such a reply effective? Rowlands (2001, p. 292) would say no, for he holds that even in case the theory is not a conceptual truth, his objection can still jeopardize it. I do not want to assess these two positions. I will limit myself to showing that even if the actualist HOT theory is construed as a conceptual thesis, it is still not circular.

Since Rowlands holds that (iii) expresses a “logical connection,” we must infer that he implicitly subscribes to the following thesis: if all X can be Y, then the concept of Y entails the concept of Y. Alternatively: if all X can be Y, then the understanding of the concept of X requires the understanding of the concept of Y. Hence Rowlands argues that since from (iii) it follows that all thoughts can be conscious, and logical connections are transitive, then the actualist HOT theory is circular. Now the question is: are we compelled to accept Rowlands’ thesis? That is: is the view that “all X can be Y” implies that the concept of X entails the concept of Y compelling? There is a strong reason to answer negatively, I take it. For one might rather embrace a view according to which a property Y of an object X is essential to X if and only if X must have Y to be what it is (see Fine, 1994). On such a view, “X can be Y” does not imply that the concept of X entails the concept of Y. Compare: just as “all bachelors could become CEOs of Apple” does not imply that the concept of bachelor entails the concept of Apple CEO (or that the understanding of the concept of bachelor requires the understanding of the concept of Apple CEO), “all mental states could become conscious” does not imply that the concept of mental state entails the concept of consciousness (or that the understanding of the concept of mental state requires the understanding of the concept of state consciousness).

At this point, Rowlands could acknowledge that his view about concept entailment is disputable, but insist—quite strangely, in my view—that (iii) would still render Rosenthal’s theory circular. To this one could reply that Rowlands is pitching the standards of informativeness too high. Consider the relational analysis of *being a parent*: X is a parent just in case there is some Y, distinct from X, such that X is either a father of Y or the mother of Y. Now, Y could certainly become a parent on his turn. Does this render the definition circular? I believe it does not. If you find the foregoing example questionable, then consider also the notorious reductive analysis of *knowledge*: *p* is known just in case *p* is the object of a justified true belief.^13^ Now, beliefs could be known as well (indeed, we are capable of self-knowledge). Does this render the definition circular? I believe that it does not. And it is reasonable to presume that on Rowlands’ standards, too many definitions would turn out to be circular.

## The first epistemic objection

The actualist HOT theorist claims that our states are conscious just in case we have a thought to the effect that we, ourselves, are in those states. The theory does not say that our higher-order thoughts must be conscious too. On the contrary, the actualist HOT theorist holds that most of our higher-order thoughts are unconscious. And that a higher-order thought T is unconscious means, of course, that we do not have a thought T* to the effect that we, ourselves, are in T. How is the actualist HOT theorist driven to this position? According to Goldman (2002, p. 118), the reasons are two: the threat of infinite regress, and the evidence that we rarely reflect on our mental states^14^—where the first reason is regarded by Goldman as the main one.^15^

Whatever the reason is, Goldman (2002, p. 118) has a problem with the claim that an unconscious higher-order thought can make something conscious. He not only considers the relevant claim “quite counter-intuitive,”^16^ but he also argues that by allowing higher-order thoughts to be unconscious, the actualist HOT theory turns out to be unable to account for the “distinctive epistemic position” we are in with respect to our own conscious mental states. The “distinctive epistemic position” Goldman has in mind is expressed by the following thesis: whenever we have a thought to the effect that we, ourselves, are in a certain mental state M, we “automatically have good evidence for believing” that M *is conscious*—where “having good evidence for believing that *p*” can be replaced by “being in a good epistemic position to tell that *p* is true.” Goldman (2002, p. 118) maintains that a theory of state consciousness ought to accommodate the fact that we are in the foregoing epistemic position, and contends that the actualist HOT theory does not do that. He writes:

When you are in a conscious mental state M—at least a salient, nonfringy conscious mental state—then you automatically have *good evidence* for believing that this state is conscious. If you are consciously thinking about Vienna, for example, that very thinking gives you excellent evidence for the proposition that this thought is conscious. […] How does the HOT theory make sense of the fact that one automatically has good evidence for the fact that these states are conscious? Since what *makes* them conscious is the existence of independent, non-conscious mental states, one could only have automatic evidence for the consciousness of the first-order states if one automatically had evidence for the existence of those *non*-conscious HOTs [higher-order thoughts]. But why should such evidence automatically be available? (Goldman, 2002, pp. 118–119)

We may rephrase “automatically” with “just in virtue of the fact that,” and hence say that Goldman’s attack rests on the following premises:

(P_1_) S has good evidence for believing that S’s mental state M is conscious just in virtue of the fact that M is conscious.

(P_2_) S has good evidence for believing that S’s mental state M is conscious only if S’s thought about M is conscious.

Where P_2_ could be rephrased with “S has good has evidence for believing that S’s mental state M is conscious only if S has good evidence for believing that S has a thought about M.” Goldman concludes that it cannot be that most of our higher-order thoughts are unconscious—a conclusion that glaringly contradicts the view of the actualist HOT theorist.

Goldman (2002, p. 135 n6) makes clear that his argument has force only if the actualist HOT theory expresses a *conceptual* truth. He makes the following point: if “all *x*’s are F” expresses a conceptual truth (like “all bachelors are unmarried”), then one cannot have evidence for something being *x* without having evidence for its being F. By contrast, if “all *x*’s are F” expresses a contingent truth (like “all water is H_2_O”), then one can have evidence for something being *x* without having evidence for its being F. Indeed, it is often argued that while you cannot know that Peter is a bachelor without knowing that Peter is unmarried, you can still know that the liquid in the glass is water without knowing that water is H_2_O. Accordingly, if “S’s M is conscious” is extensionally equivalent to “S has a thought to the effect that S is in M,” then one cannot have evidence for S’s M being conscious without having evidence for S to have a thought to the effect that S is in M.

As we know, Goldman thinks that the actualist HOT theory is best understood as a definition. Hence, he also thinks that his objection cuts against Rosenthal’s view. Once again, Carruthers (2016) tries to disarm the objection by rejecting the reading of the theory. He claims that the actualist HOT theory is an “*empirical* theory.” As such, he states, it expresses a truth like “all water is H_2_O.” Thus, Carruthers’ attempt to reject P_2_ implicitly relies on the following analogy: conscious mental states are to higher-order thoughts as water is to H_2_O.^17^ But if it is so, Carruthers goes on, then we can know that our mental states are conscious without knowing that we have a thought to the effect that we, ourselves, are in those mental states. Still, one could follow a different path: instead of trying to reject P_2_, one can try to reject *P*_*1*_.^18^ Such a reply does not hinge on the denial of Goldman’s reading of the actualist HOT theory. Rather, I submit, it works even if the actualist HOT theory does express a conceptual truth.

For recall, P_1_ says that one has good evidence for believing that one’s mental state M is conscious just in virtue of the fact that M is conscious. For instance: you have good evidence for believing that your seeing red is conscious just in virtue of the fact that you are consciouslyseeing red.^19^ Is the actualist HOT theorist compelled to accept such a view? Let us look at the details of her theory.

First, on the theory, a mental state is conscious just in case one has a thought to the effect that one is, oneself, in that state. Consider a conscious seeing. Using angle brackets, we can represent the mental content of the higher-order thought as follows:

<I, myself, am seeing red>

In general, the kind of content higher-order thoughts have is the following:

<I, myself, am in M>

As can be seen, the content does not encode the phrase *“conscious*,*”* namely, the thought does not represent state consciousness. One could hold that a *third*-order thought will encode such expression. But this is not the case. Compare the following two mental contents:

<I, myself, am seeing red>

<I, myself, am thinking about my seeing red>

The first content is the content of a second-order thought, whereas the second one is the content of a third-order thought (see Rosenthal, 2005, pp. 27, 113, 292 n17, 298, 344 n13). As can be seen, neither encodes the phrase “conscious,” namely, neither represents state consciousness. Ascending the entire hierarchy of orders will not enable us to find a content with the relevant phrase, namely, a thought that represents state consciousness.

Second, on the theory whenever one has a thought to the effect that one is, oneself, in a mental state M, it *seems* to one that one is, oneself, in M. With Rosenthal’s (2011, p. 431) slogan: “A state’s being conscious is a matter of mental appearance.” For instance: whenever I have the thought with the content <I, myself, am seeing red>, it *seems* to me that I, myself, am seeing red. However, the actualist HOT theorist does *not* claim that whenever one has a thought to the effect that one is, oneself, in a mental state M, it seems to one that one is, oneself, in a *conscious* M. Alternatively: she does not claim that whenever one has a thought to the effect that one is, oneself, in M, it seems to one that M *is conscious*. The reason is precisely that on the theory the content of the higher-order thought does not encode the phrase “conscious.”^20^ Compare the following two kinds of mental content:

<I, myself, am in M>

<I, myself, am in a conscious M>

We may grant Goldman that “S’s M is conscious” is extensionally equivalent to “S has a thought to the effect that S is in M.” On this view, <I, myself, am in a conscious M> is equivalent to the following kind of content:

<I, myself, am having a thought to the effect that I, myself, am in M>

Well, since the content of higher-order thoughts does not encode the phrase “conscious,” that content cannot be equated with <I, myself, am having a thought to the effect that I, myself, am in M> either. Hence, the actualist HOT theorist does not even claim that whenever one has a thought to the effect that one is, oneself, in a mental state M, it seems to one that one is having a thought to the effect that one is, oneself, in M.

Now we may assume for the sake of argument that seemings provide us with a kind of evidence, that is to say, that seemings put us in a certain epistemic position. We may say that if it seems to one that one is, oneself, in a mental state M, then one has good evidence for believing that one is, oneself, in M (namely, one is in a good epistemic position to tell that the proposition “I, myself, am in M” is true). For example, we may say that if it seems to me that I, myself, am seeing red, then I have good evidence for believing that I, myself, am seeing red. The actualist HOT theorist may hold that true, but she may also argue along the following lines: one has good evidence for believing that one is, oneself, in a conscious mental state M only if it seems to one that one is, oneself, in a conscious M; it seems to one that one is, oneself, in a conscious mental state M only if the content of one’s mental state about M encodes the phrase “conscious.” On the theory, the content of the thought that makes a mental state conscious does not encode “conscious.” Therefore, on the theory, P_1_ does not hold. Of course, the actualist HOT theorist may maintain that if one *had not had* a thought to the effect that one is, oneself, in a mental state M, then one *could not* have good evidence for believing that M is conscious, for from this it does not follow that one is in a good epistemic position to tell that the proposition “I, myself, am in a mental state M” is true just in virtue of the fact that M is conscious.^21^

## The second epistemic objection

Since the actualist HOT theorist explains transitive consciousness in terms of representation, he holds true the proposition that all conscious mental states are represented. Call this proposition *Rep*. Kriegel (2009a, b) endorses it too, but he asks: what is the *evidential basis* for Rep? He takes into account three kinds of evidence: *phenomenological* evidence, *conceptual* evidence, and *experimental* evidence. Phenomenological evidence branches out into *direct* phenomenological evidence and *indirect* phenomenological evidence.^22^ Then he argues along the following lines: if there is any evidential basis for the proposition that all conscious mental states are represented, then this evidential basis is direct phenomenological evidence; but if it is so, then there cannot be unconscious higher-order states (or better, second-order states). In Kriegel’s words: if the only evidential basis for Rep is direct phenomenological evidence, then “all conscious mental states are consciously represented.” Call the thesis under quotation marks the *hyper-consciousness thesis*.^23^

As Kriegel’s argument against the actualist HOT theorist is basically an argument by elimination, it is possible to block it by showing that at least one evidential basis different from direct phenomenological evidence is available. For example: if conceptual evidence turns out to be an available evidential basis for the relevant proposition, then Kriegel’s argument falls flat.

A first way to block the argument is precisely to make the move I have just mentioned: there is no need for phenomenology, one could say, for the link between state consciousness and representation is guaranteed conceptually.^24^ Along these lines, one could conceive of Rep as structurally akin to “all bachelor are unmarried” or “all vixens are female foxes” (where “conscious mental state” would be to “represented” as “bachelor” is to “unmarried” or as “vixen” is to “female fox”). What about the view of the actualist HOT theorist? The answer is not plain: on the one hand, Rosenthal explicitly denies that the actualist HOT theory expresses a conceptual truth; on the other hand, such a statement does not rule out that some theses that ground the theory are conceptually evident. For one could deny that the link between *state consciousness and higher-order thoughts* is conceptual while still maintaining that the link between *state consciousness and representation* is conceptual. At any rate, what matters here is that the actualist HOT theorist allows herself to rely also on introspective input (see Rosenthal, 2005, pp. 9, 130; Weisberg, 2020, p. 439). Moving from this suggestion, I will try to show that *indirect* phenomenological evidence is an available evidential basis for Rep*.* Clearly, assessing whether indirect phenomenological evidence is an available evidential basis for Rep makes sense only if one models Rep on non-conceptual truths such as “all ravens are black.” On this model, we have that “conscious mental state” is to “represented” as “raven” is to “black.”

First of all, let us consider Kriegel’s conditional: he says that if the only evidential basis for Rep is direct phenomenological evidence, then all conscious mental states are consciously represented. Let us have a look at his distinction between direct and indirect phenomenological evidence. We can state it as follows (see especially Kriegel, 2009b, p. 124):

**Direct phenomenological evidence.** If S has direct phenomenological evidence for the proposition that all *x*’s are F, then S “sees” each and every *x* (and “sees” that each and every *x* has F).

**Indirect phenomenological evidence.** If S has (only) indirect phenomenological evidence for the proposition that all *x*’s are F, then S does not “see” each and every *x*.

With a classical example: if S has direct phenomenological evidence for the proposition that all ravens are black, then S sees each and every raven (and sees that each and every raven is black). If S (only) has indirect phenomenological evidence for that proposition, then S does not see each and every raven. Analogously, S has direct phenomenological evidence for the relevant proposition only if S “sees” (i.e., is conscious of) all her higher-order states (or better, all her second-order states).

Bearing this in mind, let us come back to the actualist HOT theorist. As Kriegel aptly reports, she distinguishes between *typical* cases and *non-typical* cases: in typical cases, first-order states are represented by unconscious second-order states, whereas in non-typical cases first-order states are represented by conscious second-order states. Hence, the actualist HOT theorist denies that all conscious mental states are consciously represented. For the actualist HOT theorist, conscious mental states are consciously represented only when we introspect^25^ As Rosenthal puts it:

Introspection is the special case in which that HOT [higher-order thought] is conscious, which happens when a yet higher-order thought occurs—a third-order thought about the second-order thought. (Rosenthal, 2005, p. 113)

Now, in order to deal with Kriegel’s critique, one has to assume that introspecting is something more than merely having a third-order thought; one has to conceive of introspection as something that enables us to find the properties of our conscious states (see, e.g., Kriegel, 2009a, p. 364). In such a framework, just as the biologist arrives at “all ravens are black” by induction, the actualist HOT theorist could arrive at Rep *by induction*. Her inductive inference could be thus (let “ → ” be a symbol for induction):

(Inf_1_) *“All introspected first-order conscious Ms are represented”* → *“All first-order conscious Ms are represented”*

Kriegel’s (2009b, p. 119) attack is directed precisely towards the validity of Inf_1_. First, he argues that the inductive sample of the actualist HOT theorist is biased, for “what makes a conscious mental state belong to the sample is that that mental state is represented in introspection.” He makes this point also in the following impressionistic terms: “the introspecting itself constitutes the representing” (see also Kriegel, 2009a, p. 367). Second, he argues that if the inductive inference is warranted for Rep, then it is warranted for the hyper-consciousness thesis as well, for “all the instances in the sample are consciously represented” (see also Kriegel, 2009a, p. 367 n20). Is Kriegel’s critique compelling? Let us address his sub-objections one by one.

Concerning the first sub-objection, Levine (2010) and Van Gulick (2012) claim that there is no reason to think introspection is responsible for “introducing” the second-order mental state.^26^ In particular, Van Gulick remarks that the structure of Inf_1_ is the same as the structure of any scientific inference. In the following, I will show that the Levine-Van Gulick intuition is not only correct but also resists Kriegel’s reply to it. This latter is given in the following passage:

Yet the fundamental flaw in the inductive argument under consideration is not just that it proceeds from the observed to the unobserved, but that the property projected through it pertains precisely to being observed. Compare: one can justifiably infer from the fact that all observed swans are white that all swans are white, but one cannot justifiably infer from the fact all observed swans are observed that all swans are observed; from the fact that all observed swans are at an eyeshot from an observer that all swans are at an eyeshot from an observer; from the fact that all observed swans are perceptually represented that all swans are perceptually represented. The problem with the inference from the fact that all introspected conscious mental states are represented to the fact that all conscious mental states are represented is that it is structurally akin to these obviously fallacious inferences.^27^ (Kriegel, 2012, p. 479)

Hence, for Kriegel the inference made by the actualist HOT theorist is not akin to the following:

(Inf_2_) *“All observed ravens are black”* → *“All ravens are black”*

but rather to the following:

(Inf_3_) *“All observed ravens are observed”* → *“All ravens are observed”*

where the observation is to the ravens as the introspection is to conscious mental states. Now our question is: is the inference made by the actualist HOT theorist really *like* Inf_3_? Or better still: do we have any reason to say that the inference made by the actualist HOT theorist is *not like* Inf_2_? Let us see whether it is the case.

By introspecting we find that conscious first-order mental states have the property “being represented.” But represented by what? An answer is to say that by introspecting we find a second-order mental state which represents a first-order mental state. More precisely: by performing an introspection directed to a non-introspective mental state, we do not find the relation between a *third-order state and a second-order state*, but rather the relation between a *second-order state and a first-order state*. Why should we think that this answer is not viable? It seems that nothing prevents the actualist HOT theorist to give such an answer. He can say that what makes a first-order conscious mental state M belongs to the sample is not that M is represented in introspection, but rather that M is represented by a second-order mental state. Indeed, in the actualist HOT theorist’s framework, introspected first-order mental states are represented by the introspective state, but they are not represented only by the introspective state, for they are already represented by second-order mental states as well. If this is true, then the actualist HOT theorist does not project the property “being observed,” but rather the property “being represented by second-order mental states.” In Kriegel’s impressionistic words: the introspecting constitutes the representing of second-order mental states, but it does not constitute the representing of first-order mental states.^28^ We can thus reformulate the inference made by the actualist HOT theorist in more fine-grained terms:

(Inf_4_) *“All introspected first-order conscious Ms are represented by second-order Ms”* → *“All first-order conscious Ms are represented by second-order Ms”*

To put it briefly, Kriegel’s first objection is plagued by an erroneous conflation of two orders of representation.

Now consider the second sub-objection. Recall that Kriegel says that since “all the instances in the sample are consciously represented,” one may inductively infer thus:

(Inf_5_) *“All introspected first-order conscious Ms are represented by conscious second-order Ms”* → *“All first-order conscious Ms are represented by conscious second-order Ms”*

It would be as though we were making the following inference:

(Inf_6_) *“All observed ravens are black”* → *“All ravens are black and observed”*

Where one may replace “raven” with “conscious mental state,” “observed” with “introspected” and “black” with “represented.” Inf_6_ would be warranted only in case, whenever we observe a raven, we find both the property “black” and the property “being observed.” By the same token, Inf_5_ would be warranted only in case, whenever we introspect, we find both the introspective state (or better, the property “being introspected”) and the second-order mental state (or better, the property “being represented by second-order mental states”). But on the actualist HOT theorist’s model, this is simply not the case. Therefore, the actualist HOT theorist can say that the inference from “all introspected first-order conscious mental states are represented” to Rep is warranted without being forced to say that the inference from “all introspected first-order conscious mental states are represented” to the hyper-consciousness thesis is warranted.

Clearly, Kriegel’s set of base cases is larger than the actualist HOT theorist’s one, for the former consists of all his second-order mental states. But of course, we cannot identify an evidential basis for a proposition only because it is the strongest one, for our question is precisely whether we “see” (i.e., are conscious of) each and every instance of second-order mental state. Compare: we cannot say that we observe each and every raven only because this would make “all ravens are black” safer, for the simple reason that our question is precisely whether we observe each and every raven.

## The intimacy objection

The intimacy objection is arguably the most pressing objection against the actualist HOT theory: it has been raised and elaborated by some scholars (see Neander, 1998; Levine, 2001; Kriegel, 2003) and over the years has been cited by other scholars as a strong motivation for abandoning higher-order theories in favor of alternative views (see, e.g., Brook & Raymont, 2006; Horgan et al., 2006; Kriegel, 2009b; Block, 2011).

The objectors charge that the actualist HOT theory does not accommodate the “intimacy” we have with our own conscious mental states (see McClelland, 2020, p. 462; the term “intimacy” comes from Levine, 2001, Chap. 6). The intimacy thesis can be put as follows: whenever we are conscious of a mental state M, we are in M. In other words, in being in a higher-order state we err neither with respect to the *properties* of the lower-order state (this is called “qualitative intimacy”) nor with respect to the *very existence* of the lower-order state (this is called “existential intimacy”). Briefly, the intimacy thesis might be put as follows: *inner misrepresentation cannot occur* (see also Rosenthal, 2018, p. 53). In the terms of the actualist HOT theory: whenever we have a thought to the effect that we, ourselves, are in a mental state M, we are in M. For instance: whenever we have a thought with the content <I, myself, am seeing red>, we are seeing red.

Why the actualist HOT theory would not accommodate intimacy? The objection runs thus (see McClelland, 2020, p. 463; Rosenthal, 2018, p. 54): if the higher-order state neither is identical nor belongs to the lower-order state, then we *can* err both with respect to the properties of the lower-order state and with respect to the very existence of that state (i.e., inner misrepresentation can occur). As the actualist HOT theorist holds that the higher-order state neither is identical nor belongs to the lower-order state, on that theory we can err both with respect to the properties of the lower-order state and with respect to the very existence of that state. Therefore, the actualist HOT theory does not accommodate intimacy.

What are the options for the actualist HOT theorist? Basically two: either to deny that there is something like intimacy or to find a way to accommodate intimacy without abandoning her intuition—i.e., that the higher-order state neither is identical nor belongs to the lower-order state (claim (c) above).

As for the first option, Rosenthal (2011) makes a suggestion. Consider the following two theses:

(T_1_) Whenever we are conscious of a mental state M, we are in M.

(T_2_) Whenever we are in a mental state M, we are conscious of M.

Every higher-order theorist agrees that T_2_ is untenable. Now, according to Rosenthal (2011, p. 433), since T_2_ is untenable, we have “no reason to insist” that T_1_ is tenable. In his words: if it is true that “mental reality is to some extent independent of mental appearance”—unconscious states *do* occur—, then we have no reason to insist that mental appearance is dependent on mental reality—false higher-order states *do* occur. We rather “must” deny T_1_.

Is Rosenthal’s (2011) suggestion satisfying? It lends itself to two readings. First, Rosenthal is saying that the implausibility of T_2_ provides us with *a reason against* T_1_. However, one may reply that having an argument for or against the occurrence of unconscious states does not amount to having an argument for or against the occurrence of false higher-order states (and vice versa). The reason is simply that we are dealing with *different* (to be precise: *converse*) dependence relationships. On this reading, the suggestion is simply off the mark. On the second reading, Rosenthal is saying that the implausibility of T_2_ makes T_1_ *groundless*. We have no reason to hold the intimacy thesis to be true, or so the argument goes. However, this would leave the actualist HOT theory under the threat of the intimacy objection, for if the intimacy thesis turned out to be plausible, the actualist HOT theorist might still be asked to account for intimacy. On this reading, the suggestion is weak: to make the theory immune to the relevant threat, one has to show something more, namely, that T_1_ is *untenable.* Rosenthal (2005, 2011, 2018) tries to do that as well. He attempts to show that there is nothing like intimacy. He points at cases that, to his eyes, are counter-examples to the intimacy thesis.^29^

I do not want to critically assess Rosenthal’s counter-examples. Other scholars have already asked themselves whether Rosenthal’s cases admit of an interpretation on which no inner misrepresentation occurs (see, e.g., Kirkeby-Hinrup, 2014, 2016). I rather want to explore the second option. Rosenthal (2018, p. 53) does that too. He reasons as follows. Suppose there is something like intimacy. The actualist HOT theorist can accommodate it while still maintaining that the higher-order state neither is identical nor belongs to the lower-order state. He can do that by simply *“adding a provision”* to the theory. The provision precisely says that inner misrepresentation cannot occur. Such a move is supposed to satisfy the objector for the following reason: if it is true that the intimacy thesis is validated on independent grounds (for its friends: on *introspective* grounds; see especially Kriegel, 2009b; McClelland, 2020), why should a theory of intransitive state consciousness accommodate it *on his own*? In other words: if the intimacy issue is indeed independent of the question “what is it for a mental state to be conscious?”, why should a theory of state consciousness accommodate intimacy *on his own*? In Rosenthal’s words:

Still, though higher-order theories do not on their own rule out such misrepresentation, they also do not imply that it can, or ever does, occur. So if one did have suitable independent reason to think that it [= inner misrepresentation] cannot or never does occur, one could simply add that onto a higher-order theory without any problem. What is important here is that adding such a provision onto a higher-order theory would doubtless not satisfy those who use misrepresentation by consciousness as an objection to such theories. The question is why not. Adding on such a provision, especially if it were based on a suitable independent reason, delivers they [*sic!*] result the objectors ostensibly want. What more can they reasonably demand? The quick answer is that these objectors regard no theory as satisfactory unless it rules out misrepresentation on its own, without any added provision. But that just pushes the question back one step. If there is independent reason to rule it out, why must a theory do so on its own, without appeal to any added provision? (Rosenthal, 2018, p. 53)

Can Rosenthal’s reply satisfy the objector? Rosenthal is certainly right in pointing out that the intimacy issue goes *beyond* the core aim of the actualist HOT theory (where the core aim is to answer the question “what is it for a mental state to be conscious?” or “what is the difference between conscious and unconscious states?”).^30^ Still, the actualist HOT theorist does commit herself to claims that go beyond the core aim of the theory, that is, (b) and (c). It is *(c)* that the friend of intimacy targets. Indeed (let M_2_ stand for the higher-order state and let M_1_ stand for the first-order one), friends of intimacy ask to provide them with a *model of the relationship* between the M_2_ and M_1_. Now, the actualist HOT theorist limits himself to saying that M_2_ is neither identical nor belongs to M_1_, whereas intimacy involves a “tie,” i.e., a *dependence* relationship between M_2_ and M_1_. As the model of the actualist HOT theorist does not reflect such a dependence, that model does not accommodate intimacy. To sum up, Rosenthal’s attempt to accommodate intimacy is unsatisfactory in that he neglects to provide a model of the relationship between the higher-order and the lower-order state that satisfies the intimacy thesis.

Friends of intimacy are right on what follows: a model that *just* says that M_2_ is distinct from M_1_ or that even says that there is *no* dependence relationship between the two does not accommodate intimacy. Still, it seems that friends of intimacy make a further move; it seems that they claim that the *only* model that can accommodate intimacy is the following model:

(~c) Either M_2_ is identical to M_2_ or M_2_ belongs to M_1_.

This is, in fact, the *self-representationalist* model (see Kriegel, 2009b; McClelland, 2020)—what Rosenthal refers to as “the same-order theory.” On such models, higher-order states either are *identical* to the lower-order ones or are *proper parts* of them.

Now our question is: is it true that the only model that accommodates intimacy is (~ c)? (granted, merely for the sake of argument, that it does accommodate intimacy).^31^ The answer is negative, I submit. The higher-order theorist can accommodate intimacy while still maintaining that M_2_ is neither identical nor belongs to M_1_. He can speculate as follows:

(c+) M_1_ belongs to M_2_.

On such a model, M_2_ is neither identical nor belongs to M_1_, for a proper part of *x* cannot be identical to *x* (by the definition of proper parthood) and distinct things cannot be part of each other (by mereological anti-symmetry).^32^ Crucially, M_2_ cannot occur without M_1_’s occurring. This follows from a thesis that is not only intuitive but which has also been explicitly accepted by two renowned philosophers of mind: I refer to Brentano (1995, p. 71) and Husserl (2001, p. 21). According to this thesis, often referred to as “mereological essentialism” (Simons, 2003), the existence of *x* implies the existence of all of the proper parts of *x*.^33^Mereological essentialism is not uncontroversial. Still, it is a lively option, one which also has a venerable tradition.^34^

In conclusion, the actualist HOT theorist is in a position to reject the intimacy objection: even granting that there is something like intimacy, a model is available that at the same time preserves her intuition about the relationship between higher-order and lower-order states and accommodates intimacy.

## Results

Overall, I have argued that four objections leveled at the actualist HOT theory are weak. I have named them the circularity objection, the first epistemic objection, the second epistemic objection, and the intimacy objection respectively. The first two objections turn out to be weak even on a conceptual reading of the theory. The circularity objection is weak because it is based either on a highly disputable view of concept entailment or a too demanding standard of informativeness (Sect. 3), whereas the first epistemic objection is weak because the actualist HOT theorist may reject the thesis that when one’s mental state is conscious one automatically has good evidence for believing that that state is conscious (Sect. 4). As for the second epistemic objection, it is weak because indirect phenomenological evidence is an available evidential basis for the proposition that all conscious mental states are represented (Sect. 5). Finally, the intimacy objection is weak because a model is available that at the same time preserves the actualist HOT theorist’s intuition about the relationship between higher-order and lower-order states and accommodates intimacy, if any (Sect. 6). The actualist HOT theory may have some flaws, but it does not have the flaws that the aforementioned objections purport to detect.

## Footnotes

1 For a survey, see Carruthers (2016).

2 As it is raised by different authors, this objection comes in varying forms. I will rely on the form that is present in Rosenthal (2018, p. 54) and McClelland (2020, p. 463), which is meant to be the standard form.

3 For instance, Gottlieb (forthcoming) writes: “HOT theory is often framed in *relational* terms, where, for any first-order perceptual state M of a subject S, M is […] conscious at *t* iff S harbours a suitable HOT M* that represents M at *t*.” It is not clear what would make a HOT theory “relational.” Sometimes it seems it is the fact that the theory says that a *relation* between the higher-order state and the first-order state always obtains (see Brown 2015, p. 1785; Gottlieb forthcoming). But the original formulation of the theory does *not* say that. Sometimes it seems it is the fact that the theory says that the *existence* (viz. *occurrence*) of the first-order state is a necessary condition for its being a conscious state (see Brown 2015, p. 1792). But again, the original formulation of the theory does *not* say that. As the original formulation is the formulation that Rosenthal still holds (see the quotation below), there seems to be no reason to regard the latter as “relational.” Thanks to David Rosenthal for a useful exchange on this point.

4 For instance, Gottlieb (forthcoming) formulates the actualist HOT theory as follows: “*For any subject S*, S’s being in a suitable higher-order mental representation M* (an assertoric and *de se* HOT) is necessary and sufficient for *S’s being phenomenally conscious*.” (emphasis mine).

5 I limit myself to saying what follows: the issue seems to be that there is a tension between the original formulation of the theory, the possibility of false higher-order thoughts, and the assumption that awareness is factive. However, it is clear from Rosenthal’s writings that he deliberately uses “aware of” and “conscious of” in a non-factive sense (see especially Rosenthal 2005, p. 112 n12). In this paper I will stick to this use. In any case, to resolve the tension it is sufficient to amend the theory as follows: one’s mental state is conscious if and only if one has a *veridical* thought to the effect that one is in that mental state.

6 In the literature state consciousness is often said to admit of two variants: if there is “something it is like” to be in a mental state, then we speak of *phenomenal* consciousness; if the content of that mental state is available for various cognitive operations, then we speak of *access* consciousness (see Block 1995). As for Rosenthal, it seems that he simply rejects the distinction at issue (see Rosenthal 2005, p. 192; Wilberg 2010, p. 625).

7 Note that this is a simplified formulation of the theory, for if one looks at Rosenthal’s work (notably Rosenthal 1986), one finds at least three further conditions for a state to be conscious: the *causal* condition, according to which the lower-order state causes the higher-order thought; the *immediacy* condition, according to which the higher-order thought is arrived at non-inferentially; and the *simultaneity* condition, according to which the higher-order thought is (roughly) contemporaneous to the lower-order state. The causal condition has been retracted by Rosenthal (1993, p. 218 n16), and none of these conditions is relevant for current purposes. This is why I dare to work with a simplified formulation.

8 Carruthers (1996, p. 165) seems to present his higher-order theory as a *recursive* one. However, Carruthers (2016) seems to hold that higher-order theories are reductive in nature.

9 In the following I assume the common (and perhaps naïve) view on circularity, one on which the definition of *x* by means of *y* is circular if and only if the concept of *x* entails the concept of *y and *vice versa.

10 Again, if one holds “all thoughts are conscious” to be a result of observation, then one will face a problem of infinite regress (of thoughts), not a problem of circularity.

11 For instance, one could say that *x* is a thought if and only if *x* has an intentional object. I am not endorsing this view, but rather merely pointing out that other definitions are available.

12 Actually, Rowlands (2001, p. 297) puts forwards another “candidate” besides (iii), but this detail is not germane to my discussion. Note that Rowlands (2001, p. 295–298) usage of “essentially” is not always calibrated.

13 While in the previous case the analogy was conscious mental state: higher-order thought = parent: child, here the analogy is conscious mental state: higher-order thought = knowledge: belief. It is implicit that one can define belief without referring to knowledge. For instance, one may say that *x* is a belief if and only if *x* is a mental state with assertoric attitude.

14 For Rosenthal (2005), higher-order thoughts become conscious only when we *introspect*. More precisely, a higher-order thought is conscious just in case we have a *third-order* thought, i.e., a thought about the thought to the effect that we, ourselves, are in a certain mental state. More on this in §5.

15 Yet, when one looks at Rosenthal’s works (especially Rosenthal 2005, p. 184), it seems that the actualist HOT theorist arrives at the relevant claim by observing that we rarely engage ourselves in introspection. On this basis, she denies that her theory is threatened by the infinite regress problem.

16 The idea that an unconscious mental state can make conscious another mental state is held to be highly problematic in itself by more than one scholar (see, e.g. Smith, 1986, p. 150; Rowlands, 2001, pp. 304–305). Still, others do not see anything wrong with it (see, e.g., Aquila, 1990, p. 81; Byrne, 1997, pp. 107–108). I concur with the latter.

17 One might wonder whether the advanced analogy is convincing. For one could contend that it is more natural to say that water is to H_2_O as state consciousness is to the *neural substrates* (viz. the cortical regions) associated with it. This would not amount to denying that the actualist HOT theory can receive experimental support (on this, see Rosenthal 2005, p. 229; Weisberg 2020, pp. 450–452).

18 Carruthers (2016) subscribes to P_1_. He says that whenever I have a conscious pain, I “surely know that the pain is conscious.” In the following I will show that the actualist HOT theorist is not compelled to accept such a thesis.

19 Following Kriegel (2009b, pp. 25, 26 n7, 32), I use the suffix “-ly” in a way that “Mary consciously thinks of *x*” is equivalent to “Mary’s thought of *x* is conscious.” Thus, in such a framework “Mary consciously represents her first-order state” will be equivalent to “Mary’s second-order state (about her first-order state) is conscious.”.

20 If consciousness were an *intrinsic property* of mental states, then it might be that by having a thought to the effect that one is, oneself, in M, it seems to one that M is conscious (assuming that having a thought about *x* means having a thought about all the intrinsic properties of *x*). And yet on the actualist HOT theory this is *not* the case. See especially Byrne (1997, p. 107) on that.

21 A related objection raised by Goldman (2002, pp. 118–119) is that it is not clear how unconscious higher-order thoughts explain why we usually *report* our conscious mental states as being conscious. As Rosenthal (2019, p. 195) rightly notes, though, the objection fails simply because we do *not* usually report our conscious mental states as being conscious—even if we are in them. And an explanation of this might be that the contents of our higher-order thoughts do not represent state consciousness.

22 To tell the truth, Kriegel considers evidence *from philosophical principles* as well, but I think that this latter can be equated to some degree with conceptual evidence. See also Kriegel (2012, pp. 478–479) on that.

23 One might legitimately ask why I do not adopt Kriegel’s name for this thesis, which is actually “ubiquity of inner awareness thesis” (Kriegel 2009b, p. 181). The reason is strictly practical: it is because I have found that Kriegel’s name is used in the literature equivocally. For example, Levine (2010) and McClelland (2015, p. 2470) call “ubiquity thesis” what Kriegel (2009b, p. 300) would refer to as “awareness thesis.”.

24 This move seems to be made for instance by Lycan (1996).

25 According to Kriegel’s reconstruction of the actualist HOT theorist’s model of introspection, we have that when a subject S represents a second-order mental state (i.e., when S is in an introspective mental state), then S represents the first-order mental state as well. For example: when I represent my state about my seeing of the sky, then I represent the seeing of the sky as well. This is why Kriegel can write that in introspection first-order conscious mental states are consciously represented. Even though in this context I buy Kriegel’s reconstruction, I still harbour doubts as to its faithfulness. In effect, in some footnotes Rosenthal (2005, pp. 292 n17, 344 n14) points out that his concept of introspection “short circuits the explicit hierarchy” of lower-order mental states: he claims that in introspecting an introspection—if you want: in having a *fourth*-order mental state—it is unlikely that we represent the relevant second- and first-order mental states. On the contrary, it is very likely that that we simply represent the third-order one. One might thus expect that this apply to third-order mental states as well.

26 Actually, Levine (2010) arrives at this conclusion by arguing that what we are conscious of in introspection is not the introspection itself, but rather the second-order mental state (and perhaps the first-order one as well). Still, it is hard to me to see how such a remark would disprove Kriegel’s first sub-objection.

27 In writing that the flaw of the argument is not *just* that it proceeds from the observed to the unobserved, Kriegel suggests that in this context what is problematic is the induction *as such*. Yet, not only is such an objection absent in his original examination of indirect phenomenological evidence but it also collides with his own way of proceeding: in effect, Kriegel (2009b, p. 123) acknowledges that he himself arrives at Rep *inferentially*, for phenomenological evidence (either direct or indirect) cannot directly support universal propositions. His inference, though, is not an inductive inference, but rather an inference to the best explanation. It is a two-step inference: first, he reasons that since all *his* conscious mental states are represented, it must the case that all *others’* conscious mental states are represented as well. Second, he reasons that since all conscious mental states of (normal adult) *humans* are represented, it must be the case that *all* (actual or merely possible) conscious mental states are represented. For an assessment of such an inference, see Mehta (2013).

28 This is why the following (inductive) inference would be unwarranted: *“All introspected second-order Ms are represented in introspection”* → *“All second-order Ms are represented in introspection.”*

29 A case which Rosenthal (2005, pp. 209, 211–212, 2011, p. 436, 2018, p. 55) often advances is the phenomenon known as dental fear: we (only) have a thought to the effect that we, ourselves, are feeling pain but we are not feeling pain; rather we are just feeling vibration. The higher-order thought seems to be caused by the feeling of vibration *plus* fear. In general, the relevant cases have the following structure: we (only) have a thought to the effect that we are in M but we are not in *M*; rather we are in *N*—where M and N are first-order mental states. Here N occurs but M does not. Moreover, M is conscious but N is not. Thus, on the theory, not every conscious mental state is a state one is in: *some* conscious mental states occur (viz. exist), *some* do not.

30 Goldman’s (2002, p. 117) formulation of the theory is thus inaccurate. It is as follows: S’s M is conscious iff S has a thought to the effect that S is in M *and S is in M*. The second condition is not stated by the actualist HOT theorist.

31 Weisberg (2008, pp. 166–179) and Rosenthal (2018, p. 51–52) argue that some self-representationalist models do not accommodate intimacy. For related discussion, see McClelland (2020, p. 476).

32 An anonymous reviewer for this journal asks how this solution differs from Coleman’s (2015) “quotational” HOT theory, which indeed seems to take M_1_ to be a proper part of M_2_ (see, e.g., Coleman 2015, p. 2727). My reply is twofold: first, my solution is different in that it does *not* employ the device of mental quotation. Second, it is different in that it does *not* abandon a representational view of transitive consciousness—i.e., the idea that if *x* is conscious of *y*, then *x* represents *y* (see §2 above). For a critique of Coleman’s theory, see Rosenthal (2018, pp. 60–64).

33 Less informally, mereological essentialism can be formulated as follows: if *x* exists and *y* is a proper part of *x*, then *y* exists. In this regard, note that (c +) does *not* contradict the claim that not all mental states are conscious—the main motivation behind higher-order theories. For from the claim that *x* is a proper part of *y* it does not follow that if *x* occurs, then *y* occurs.

34 Mereological essentialism is associated with Chisholm but was already endorsed by Leibniz (see Simons 2003, pp. 2 and 319).
